# Surgical management of warfarin-associated spontaneous bilateral subdural hematoma in a mechanical heart valve patient: a rare case report

**DOI:** 10.1097/MS9.0000000000005513

**Published:** 2026-08-12

**Authors:** Aslı Aydın Taşkoparan

**Affiliations:** Department of Neurosurgery, Düzce Atatürk State Hospital, Düzce, Turkey

**Keywords:** case report, drug effect, mechanical aortic valve, subdural hematoma, warfarin

## Abstract

**Background and importance:**

Subdural hematomas (SDH) may occur spontaneously in patients with intracranial hypotension or in those taking anticoagulants such as warfarin. Bilateral cases are rare and often indicate warfarin-induced vascular fragility.

**Case presentation:**

A 68-year-old male with a mechanical aortic valve on chronic warfarin therapy presented with dizziness and nausea and was initially diagnosed with a small left parietal subarachnoid hemorrhage, with imaging showing no vascular anomaly; it remained stable, and he was discharged. Forty days later, he was readmitted with dizziness and confusion, and imaging revealed bilateral acute SDH. Warfarin was discontinued and low-molecular-weight heparin was initiated, but rapid hemorrhage progression necessitated bilateral burr-hole evacuation, followed by reoperation for a recurrent right parietal hematoma. Anticoagulation was carefully adjusted due to the competing risks of valve thrombosis and rebleeding. Postoperatively, he developed transient hemiparesis that resolved, and subsequent ventricular dilatation was managed with lumbar puncture.

**Clinical discussion:**

Warfarin, widely used for cardiovascular disease, increases the risk of bleeding complications, including subdural hematoma, especially in patients with mechanical heart valves. Patients and clinicians are confronted with both clinical and surgical challenges.

**Conclusion:**

Subdural hematoma remains a challenging condition to manage. Timely surgical intervention might be necessary and life-saving, even while on warfarin therapy. Once the hemorrhage is controlled, anticoagulation can be safely resumed.

## Introduction

Subdural hematomas (SDH) result from bleeding between the dura mater and the arachnoid membrane, are usually associated with head trauma, and can occasionally occur spontaneously in patients diagnosed with spontaneous intracranial hypotension or in those using anticoagulant medication^[^1^]^. The bilateral occurrence is relatively rare and suggests a more diffuse process of warfarin-induced vascular fragility or coagulopathy^[^2,3^]^.

Warfarin is a widely used anticoagulant for the prevention of thromboembolic events in patients with cardiovascular diseases. Although its efficacy in preventing stroke and systemic embolism is well-documented, warfarin also carries the risk of bleeding complications, including intracerebral, subdural, or subarachnoid hemorrhage (SAH)^[^4^]^. In patients using anticoagulants, minor trauma or even spontaneous bleeding can lead to a significant accumulation of blood in the subdural space. Thus, spontaneous bilateral subdural hematoma (SBSDH) is a challenging medical condition that becomes even more complex in patients on anticoagulant therapy, such as warfarin, especially those with mechanical heart valves^[^5^]^.

HIGHLIGHTS

- Timely surgical intervention can be lifesaving, even in patients on warfarin, when indicated by neurological deterioration or mass effect.
- Careful adjustment of anticoagulant therapy is crucial to balance the risks of rebleeding and mechanical valve thrombosis.
- Early recognition of postoperative complications such as hydrocephalus and appropriate management can prevent long-term neurological deficits.

Patients with SBSDH typically present with a range of neurological symptoms, including headache, nausea, vomiting, altered mental status, focal neurological deficits, and coma^[^6^]^.

In this report, we aim to explain the clinical presentation of a patient and share our experience with him. We hope that this case highlights some drawbacks of management strategies, as such cases are rarely discussed in the literature, and presents short-term follow-up for SBSDH in a patient on warfarin therapy. This case report was submitted in accordance with the Transparency in the Reporting of Artificial Intelligence (TITAN) Guidelines^[^7^]^.

## Case presentation

**Figure 1.**
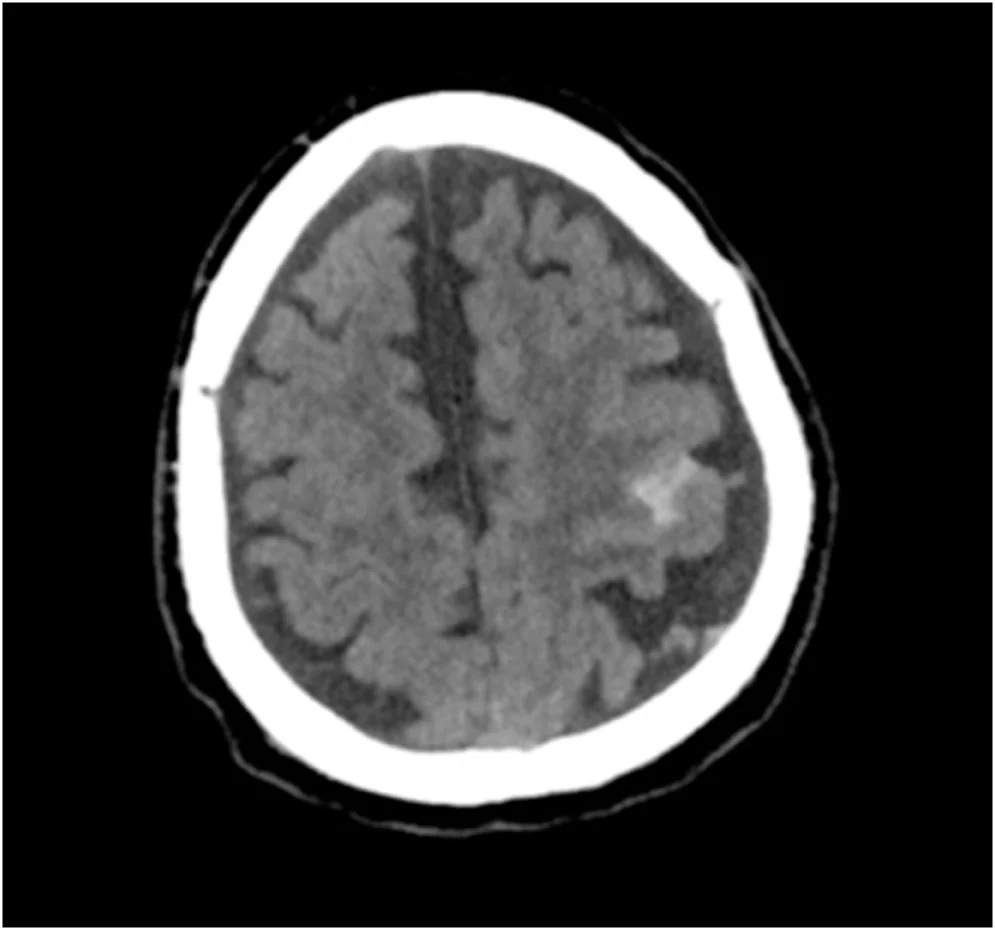
CT images of focal SAH at initial admission.

**Figure 2.**
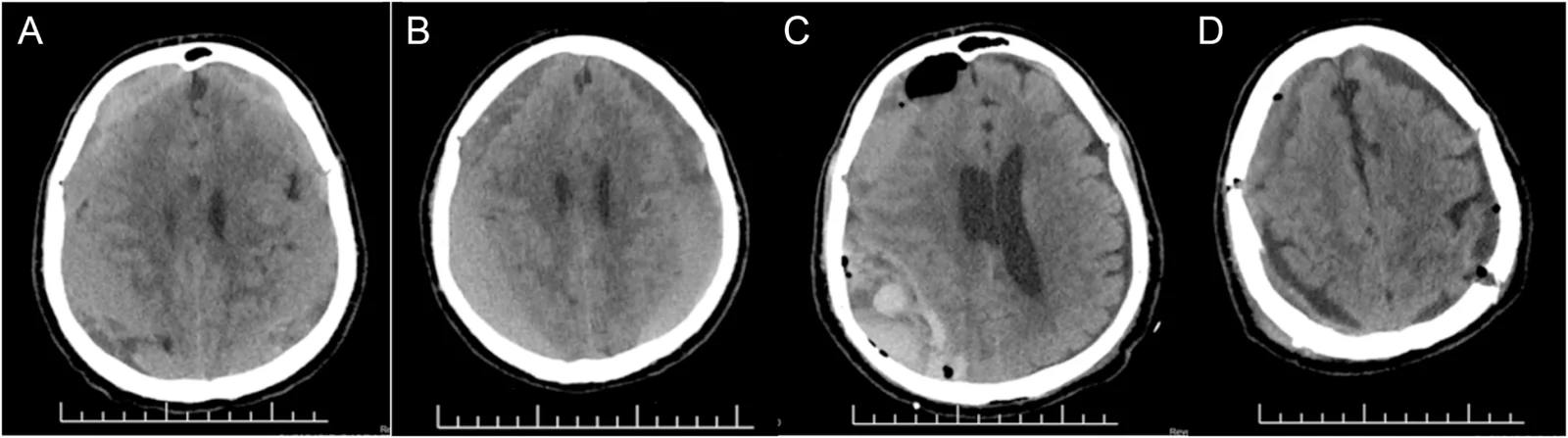
(A) Bilateral acute SDH with subacute components; (B) CT scan on the following day showing progressive hemorrhage; (C) Rebleeding area in the early post-operative period; (D) Pre-discharge imaging.

A 68-year-old male with a history of mechanical aortic valve replacement and chronic warfarin therapy presented with a 2-day history of progressive dizziness and nausea. There was no family history of medical conditions or medication-related issues. He reported no history of trauma. The physical examination revealed no evidence of fall-related head injury. The Glasgow Coma Scale (GCS) score was 15, and the neurological examination was intact. A CT scan of the head was performed, and subarachnoid hemorrhage (SAH) was diagnosed based on focal sulcal hyperdensity in the left parietal lobe (Fig. 1). Diagnostic CT angiography was performed, and no vascular anomaly was detected. During his hospital stay, daily clinical evaluations revealed no major deficits aside from a brief complaint of numbness in his right arm. After 3 days of follow-up, repeat CT scans showed no significant progression of the hemorrhage, and the patient was discharged. Forty days after his first visit, the patient was brought to the emergency department with dizziness and mild confusion, with a GCS score of 14. The initial CT scan showed bilateral acute SDH with subacute hemorrhagic components (Fig. 2A). He was admitted to the neurosurgery department for initiation of antiepileptic therapy and close monitoring of vital signs. A cardiovascular surgery (CVS) consultation was obtained regarding warfarin discontinuation, and the medication was stopped. Low-molecular-weight heparin (LMWH) was started at a dose of 40 mg/0.4 mL, administered twice daily (2 × 1), based on an estimated body weight of about 80 kg. Subsequent MRI revealed no additional findings.

The next day, a scheduled CT scan showed dramatic progression of the hemorrhage with a significant bilateral mass effect (Fig. 2B). Neurological examination revealed a mild left-sided hemiparesis. A surgical decision was made due to the patient’s neurological deterioration, loss of extremity strength, and the mass effect of the hematoma. Laboratory findings indicated an International Normalized Ratio (INR) level of 1.76. The patient was immediately transferred to the operating room, and a bilateral burr-hole evacuation of the hematoma was performed. Considering the patient’s significant risk of bleeding, a more controlled and less invasive surgical approach was initially preferred for hematoma evacuation, and burr-hole drainage was performed. Following surgery, the patient was admitted under close observation, and INR monitoring was coordinated by the CVS and cardiology departments. On postoperative day 2, owing to the risk of mechanical valve thrombosis, the LMWH dose was adjusted to 60 mg/0.6 mL. On postoperative day 4, during the daily visit, worsening of his left arm weakness was observed, and immediate re-imaging revealed a significant increase in the right parietal subdural collection and cortical compression, necessitating emergency reoperation (Fig. 2C). A right parietal craniotomy was performed to evacuate the hematoma.

Considering the patient’s risk of mechanical valve thrombosis and intracranial hemorrhage, the LMWH dose was readjusted to 40 mg/0.4 mL, administered twice daily (2 × 1). Echocardiography (ECHO) was performed to assess heart valve function, but it revealed no pathological findings. On day 2 following reoperation, cranial imaging showed that the risk of rebleeding had decreased, leading to another dose adjustment, with LMWH being stabilized at 60 mg/0.6 mL. On postoperative day 5, as the patient’s left-sided hemiparesis had nearly resolved, a hematology consultation was requested to investigate potential hematological causes. However, no remarkable findings were detected on hematological testing. The patient was discharged with a GCS score of 15 and a modified Rankin Scale score of 0 on postoperative day 12, with stable follow-up and neuroimaging findings (Fig. 2D).

**Figure 3.**
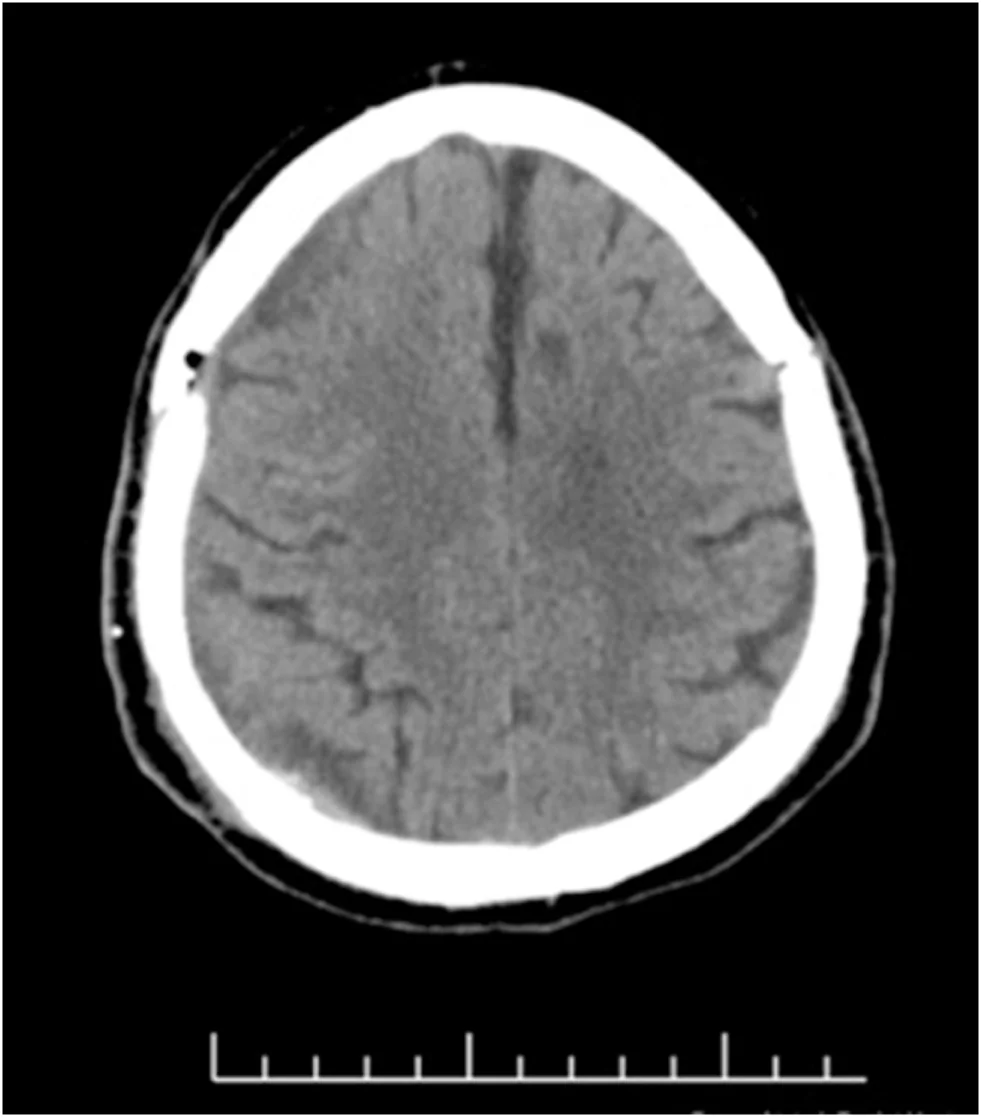
Three-month follow-up CT scan confirming the absence of recurrence or residual disease.

During outpatient follow-up, the patient developed an unsteady gait in the first month. Imaging revealed ventricular dilatation; therefore, intracranial pressure (ICP) was measured simultaneously with a therapeutic lumbar puncture. Measurement with a standard narrow-gauge manometer showed an opening pressure of 31 mmHg. Controlled cerebrospinal fluid drainage was performed, and the procedure was terminated once the ICP returned to the normal range. The patient was monitored after the procedure in the absence of any complaints, and he was discharged with a GCS score of 15 and a modified Rankin Scale score of 0. Warfarin therapy was resumed on day 20 following the last operation, under cardiology supervision. After 3 months of follow-up, the patient remained asymptomatic, and CT confirmed no recurrence (Fig. 3).

## Patient perspective

Following the stabilization of my level of consciousness and in the absence of further neurological deterioration, I was discharged with a plan for outpatient follow-up. During subsequent visits, I noticed a gradual return to my normal self. Although I occasionally experienced some imbalance when walking, it did not significantly interfere with my daily activities. The weakness in my arm had almost completely resolved, and I became increasingly confident in using it again. More importantly, I sensed a clear recovery in my thinking and awareness, which reassured me and positively influenced my quality of life.

## Discussion

The management of SBSDH in patients receiving warfarin therapy, particularly in those with mechanical heart valves, remains a significant clinical challenge because of the decision about whether to discontinue warfarin while considering the risk of thrombosis. Thus, we emphasize the importance of multidisciplinary collaboration and individualized treatment plans for managing such complex cases. A literature review revealed that there are no clear, guideline-based recommendations for the management of these patients. In addition to the rarity of such cases, the treatment of concomitant comorbidities remains debatable. The relationship between anticoagulants and SDH was examined in a comprehensive meta-analysis by Connolly et al. They reported that vitamin K antagonists increased the risk of subdural hematoma by approximately threefold compared with other antiplatelet agents^[^4^]^.

To the best of our knowledge, the largest clinical series of warfarin-associated intracranial hemorrhages was reported by Kawamata *et al*. In their 27-patient series, subdural hematoma was observed in approximately 51% (14 patients), of whom 85% (12 patients) required surgical intervention. Similar to our case, the majority of their patients had mechanical heart valve prostheses (74%) and could discontinue anticoagulant therapy for an average of only 3 days. Despite these precautions, one patient with a mechanical valve prosthesis experienced thromboembolic complications. However, the study emphasized that resumption of anticoagulation therapy in the early postoperative period did not lead to recurrent hemorrhage in any patient^[^8^]^. Similarly, in our case, warfarin was restarted under cardiology supervision on postoperative day 20 in a controlled manner, and no complications have been observed to date.

SBSDH has been reported to be associated with spontaneous intracranial hypotension (SIH) in approximately 10% of cases^[^9^]^. In many cases, a coexisting clinical presentation with more dominant symptoms may overshadow the underlying SIH and potentially serve as a facilitating factor. In our case, given that the clinical findings were not compatible with SIH and that the ICP measured via lumbar puncture at 1 month was 31 mmHg, which was interpreted as a post-operative complication, anticoagulant-associated vascular fragility appears to be a more plausible explanation than intracranial hypotension. Based on these findings, two potential etiological mechanisms may be considered: a preceding SAH or an isolated warfarin-induced reduction in vascular elasticity, resulting in an increased propensity for hemorrhage. As is known, warfarin may induce uncontrolled apoptosis in vascular smooth muscle cells, which in turn may lead to vascular smooth muscle proliferation and, ultimately, vascular calcification, thereby increasing susceptibility to bleeding^[^10^]^.

Regarding the SAH detected at the patient’s initial presentation to our center, such a hemorrhage may act as a “sentinel” event, potentially triggering a cascade that culminates in more significant bleeding days or even weeks later. In our review of the literature, delayed SDHs have most commonly been reported following head trauma, whereas we did not encounter reports describing SDH developing after such a prolonged interval following SAH^[^11^]^. Although these two clinical entities could be interpreted as independent events, we argue that the sentinel hemorrhage hypothesis provides a more coherent explanation. Furthermore, it is well established that cases in which SAH coexists with SDH are typically associated with aneurysmal rupture, while spontaneous non-aneurysmal convexity SAH is more frequently linked to cerebral amyloid angiopathy (CAA)^[^12^]^. In this context, a recent large-scale study evaluating the relationship between CAA and non-traumatic SDH demonstrated that the presence of CAA increases the risk of SDH by approximately threefold compared to other cerebrovascular diseases^[^13^]^.

This point represents a clinically valuable and instructive perspective. Small hemorrhages that are initially limited and may not be detectable on early imaging or clinical evaluation can progress over time, particularly in patients receiving anticoagulant therapy due to increased vascular fragility or as a consequence of an independent risk factor such as CAA. Such progression may ultimately lead to significant clinical deterioration and even mortality. Based on these considerations, we recommend close outpatient follow-up and emphasize the importance of educating caregivers regarding the early recognition of potential neurological deterioration.

In addition to the literature review, two case reports describe patients who were managed conservatively^[^14,15^]^. Surgical options are life-saving choices when the patient’s neurological condition requires intervention; however, in patients with a stable clinical course, a conservative approach may be preferred. Although these findings provide some guidance, further evidence is needed to optimize medication adjustments and effectively manage dynamic clinical changes in cases in which a subdural hematoma develops during mandatory anticoagulant therapy.

## Conclusions

SBSDH is a rare but life-threatening complication in patients with mechanical heart valves receiving warfarin. A multidisciplinary approach is crucial for planning patient follow-ups and anticoagulant therapy dosages. Early recognition, prompt adjustment of anticoagulation, and appropriate surgical interventions are key to improving patient outcomes. Ongoing research and clinical trials are necessary to optimize the management and prognosis of this complex condition.

## Data Availability

The data supporting the findings of this study are available from the corresponding author upon reasonable request.

## Research registration unique identifying number (UIN)

Not applicable. This study is a case report and does not require registration.
